# Knockdown of anion exchanger 2 suppressed the growth of ovarian cancer cells via mTOR/p70S6K1 signaling

**DOI:** 10.1038/s41598-017-06472-w

**Published:** 2017-07-25

**Authors:** Li-Jun Zhang, Renquan Lu, Ya-Nan Song, Jian-Yong Zhu, Wei Xia, Miao Zhang, Zhi-Yi Shao, Yan Huang, Yuqi Zhou, Hongqin Zhang, Lin Guo, Meiqin Zhang, Hong Zhang

**Affiliations:** 1 Central Laboratory, Seventh People’s Hospital of Shanghai University of TCM, Shanghai, 200137 China; 2 Department of Clinical Laboratory, Fudan University Shanghai Cancer Center, Shanghai, 200032 China; 3 Department of Oncology, Shanghai Medical College, Fudan University, Shanghai, 200032 China; 4 Department of Nuclear Medicine, Seventh People’s Hospital of Shanghai University of TCM, Shanghai, 200137 China; 5 Department of Gynecological Oncology, Fudan University Shanghai Cancer Center, Shanghai, 200032 China

## Abstract

Anion exchanger 2 (AE2, encoded by SLC4A2) is a sodium-independent chloride/bicarbonate transporter and implicated in the regulation of intracellular pH and membrane potential. Previous studies have linked AE2 to the tumorigenesis of various cancers. Here, AE2 was identified as an up-regulated protein in ovarian cancer tissues compared to adjacent non-tumor lesions based on quantitative proteomics analysis. AE2 mRNA was also overexpressed in human ovarian cancer samples, and that AE2 overexpression correlated with the shortened survival time of ovarian cancer patients. Short-hairpin RNA-mediated knockdown of AE2 in A2780 and SK-OV-R3 cells inhibited cell growth and induced cell cycle G1 phase arrest. In nude mice, its stable knockdown inhibited the tumorigenicity of A2780 cells. Gene set enrichment analysis on The Cancer Genome Atlas dataset identified that the cell cycle process and mTOR pathway were correlatively with the AE2 expression. Expression of key regulators of G1/S transition (Cyclin D1 and CDK4), and phosphorylation levels of p70S6K were notably reduced in AE2 knockdown cells. Moreover, experiments with mTOR inhibitor suggested that AE2 may promote cell cycle progression through mTOR/p70S6K1 pathway. Together, our results suggest up-regulated AE2 promotes ovarian cancer tumorigenesis by activating mTOR/p70S6K1 pathway and implicate the potential application of AE2 in cancer therapy.

## Introduction

Ovarian cancer is one of the most lethal forms of reproductive system tumors and the fifth most common cause of cancer-related deaths for women in the world^1^. Although advances have been achieved in the detection and treatment of ovarian cancer, the five-year survival rate for patients with ovarian cancer is only 30%^2^. A more comprehensive understanding of the molecular mechanisms of ovarian carcinogenesis is urgently needed to develop more effective methods of the diagnosis, treatment and prevention of this disease.

Initially, we set out to identify differentially expressed proteins between ovarian cancer tissues and adjacent non-cancerous tissues by isobaric tandem mass tags (TMT) labelling and quantitative proteomics analysis. Among the identified differentially expressed proteins (differential expression ratio >2, identified peptides >3 and *P*-value < 0.05), the up-regulation of Anion exchange protein 2 (AE2) was confirmed by analyzing The Cancer Genome Atlas (TCGA) gene expression microarray datasets from ovarian serous carcinomas. AE2 (SLC4A2) is a member of the sodium-independent chloride/bicarbonate transporters which include three anion exchangers, AE1 (SLC4A1), AE2 (SLC4A2) and AE3 (SLC4A3). AEs are fundamentally important to regulate intracellular pH (pHi) and membrane potential through conducting electroneutral transmembrane exchange of chloride/bicarbonate in response to environmental stimuli^3–6^. The transmembrane domain in AEs carries out the chloride/bicarbonate exchange function and is the region with the highest sequence similarity^7^. The homeostasis of pHi is a prerequisite for normal cell function. A large number of studies have indicated that pHi homeostasis is frequently altered in cancers, including ovarian cancer^8–10^. Recent studies have proposed that AE1 can increase gastric and colon carcinogenesis by promoting cell proliferation independent of effects on pHi regulation^11–14^. AE2 was found up-regulated in hepatocellular carcinoma^15, 16^ and colon cancer^17^. In gastric cancer, AE2, regulated by gastrin, was up-regulated or down-regulated in different cancer stages^18, 19^. A single nucleotide polymorphism (SNP) located in *SLC4A2* was found associated with reduced risk of bladder cancer^20^. However, no studies have examined the clinical relevance of AEs to ovarian cancer or the potential roles in ovarian carcinogenesis.

Therefore, the present study examined the association between AE2 expression and the overall survival of ovarian cancer patients. To further explore the roles of AE2 in ovarian tumorigenesis, cell growth and cell cycle progression were assessed in ovarian cancer cells after AE2 expression was knocked down by lentivirus-mediated RNA interferance (RNAi). We further demostrated the involvement of mTOR/p70S6K1 signaling pathway. The present study provides novel insights into the roles of AE2 and the possible regulatory mechanisms during the tumorigenesis of ovarian cancer.

## Results

### AE2 is overexpressed in ovarian cancer tissues

**Figure 1:**
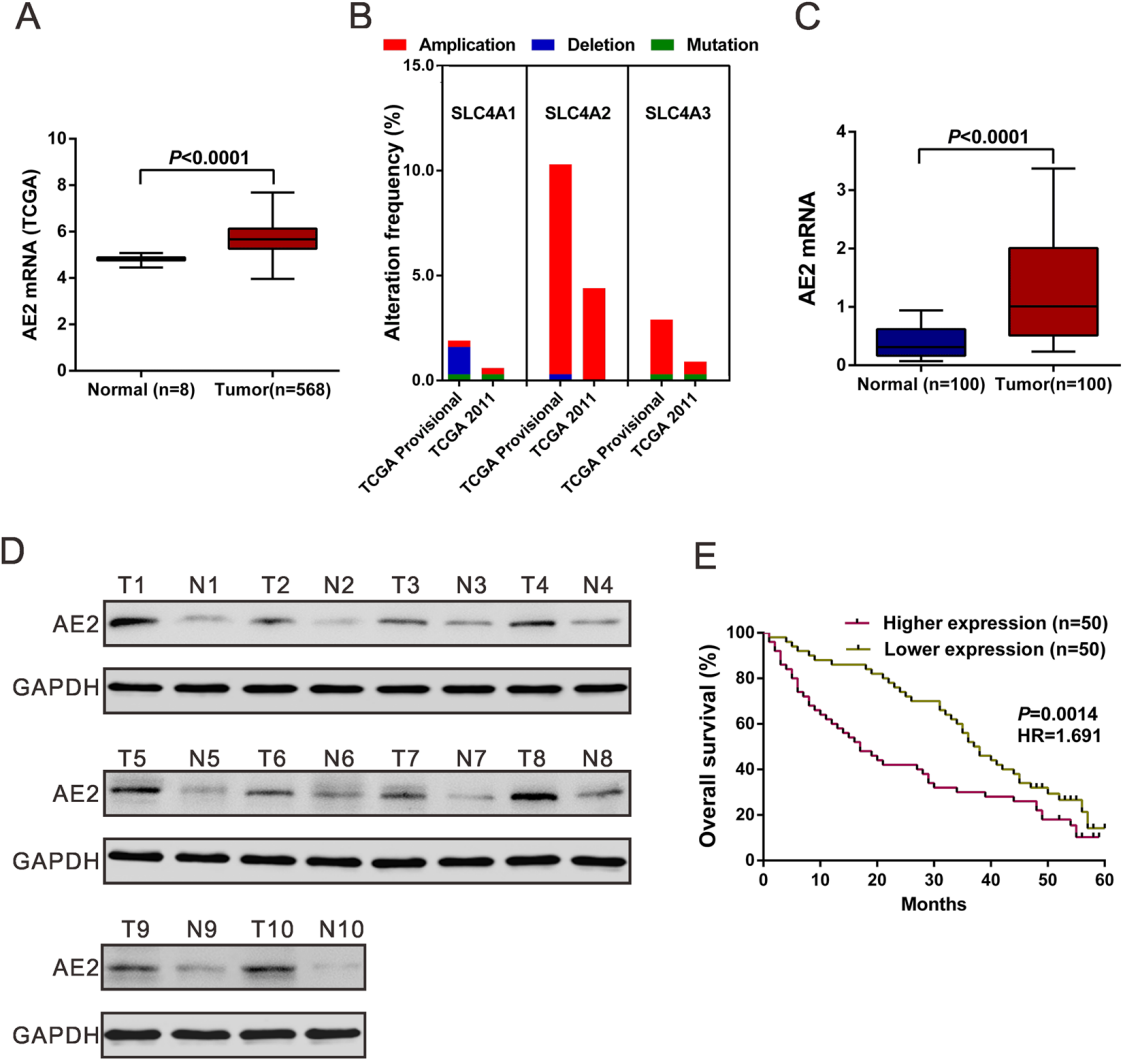
AE2 was up-regulated in human ovarian cancer tissues. (**A**) AE2 mRNA was over-expressed in ovarian cancer tissues compared to the normal tissues in TCGA dataset. Box-whiskers plot (Min to Max) was shown. (**B**) Genomic alterations of AE1 (SLC4A1), AE2 (SLC4A2) and AE3 (SLC4A3) in cBioportal ovarian cancer database. (**C**) mRNA expression of AE2 was examined by qPCR and normalized to GAPDH expression in 100 pairs of matched ovarian cancer tissues and adjacent non-tumorous tissue. Box-whiskers plot (Min to Max) was shown. (**D**) The protein levels of AE2 were higher in 10 pairs of matched samples of ovarian cancer tissue (**T**), compared with adjacent non-tumorous tissues (**N**). GAPDH was served as a loading control. (**E**) Kaplan–Meier survival analysis of 100 patients with ovarian cancer.

To discover differentially expressed proteins between ovarian cancer tissues and non-cancerous tissues, quantitative proteomics methods were applied by TMT labelling and LC-MS/MS analysis. Using a differential expression ratio cut-off of 2-fold, 190 up-regulated proteins (Table S1) and 137 down-regulated proteins (Table S2) were identified. When a minimum peptide number of 3 was set as additional selection criterion, a total of 27 differentially expressed proteins was found (13 up-regulated and 14 down-regulated) (Tables 1 and 2), among which the up-regulation of Anion exchange protein 2 (AE2) was confirmed by analyzing The Cancer Genome Atlas (TCGA) gene expression microarray datasets from ovarian serous carcinomas (Fig. 1A). These data promoted us to choose AE2 for further investigation.

**Table 1:** Proteins up-regulated in ovarian cancer tissues.

Protein Name	Accession Number	Peptides	Mann Whitney Test (P-Value)	C/N ratio
Polymeric immunoglobulin receptor	P01833	4	0.0039	2.84
ADP/ATP translocase 2	P05141	7	<0.0001	2.68
Pleckstrin homology domain-containing family A member 6	Q9Y2H5	4	<0.0001	2.58
Coagulation factor V	P12259	4	0.00035	2.41
CD9 antigen	P21926	4	0.00091	2.40
Protein-glutamine gamma-glutamyltransferase K	P22735	4	<0.0001	2.32
Lactotransferrin	P02788	6	<0.0001	2.28
Ubiquitin-like protein ISG15	P05161	5	0.0039	2.27
Poly [ADP-ribose] polymerase 1	P09874	4	<0.0001	2.14
Nucleolar RNA helicase 2	Q9NR30	5	<0.0001	2.13
Catenin alpha-2	P26232	5	0.00035	2.04
Anion exchange protein 2	P04920	4	<0.0001	2.02
Ribosome-binding protein 1	Q9P2E9	4	<0.0001	2.02

**Table 2:** Proteins down-regulated in ovarian cancer tissues.

Protein Name	Accession Number	Peptides	Mann Whitney Test (P-Value)	C/N ratio
Four and a half LIM domains protein 1	Q13642	4	<0.0001	0.29
Epsilon-sarcoglycan	O43556	6	<0.0001	0.30
Anthrax toxin receptor 2	P58335	5	0.00035	0.35
A-kinase anchor protein 12	Q02952	4	<0.0001	0.37
Arylacetamide deacetylase	P22760	5	0.00095	0.37
Glutathione S-transferase theta-1	P30711	4	0.0039	0.39
Cadherin-5	P33151	5	0.0039	0.44
Vimentin	P08670	4	<0.0001	0.44
Caveolin-1	Q03135	4	<0.0001	0.46
Phospholipid-transporting ATPase IK	O60423	4	0.0039	0.46
Alcohol dehydrogenase class-3	P11766	4	<0.0001	0.46
Neural cell adhesion molecule L1-like protein	O00533	4	<0.0001	0.47
Dynein assembly factor 1, axonemal	Q8NEP3	5	0.0039	0.49
Sterol regulatory element-binding protein cleavage-activating protein	Q12770	4	0.0018	0.49

By analyzing ovarian cancer datasets with the cBioPortal (Fig. 1B), we found the highest frequency of AE2 (SLC4A2) gene amplification (TCGA Provisional, 10.0%; TCGA 2011, 4.4%), as compared to AE1 (SLC4A1) (TCGA Provisional, 0.3%; TCGA 2011, 0.3%) and AE3 (SLC4A3) (TCGA Provisional, 2.6%; TCGA 2011, 0.6%). We then detected AE2 mRNA expression in 100 pairs of matched ovarian cancer and noncancerous tissue samples using quantitative reverse transcription PCR (qPCR) analysis. AE2 mRNA expression was significantly higher in ovarian cancer tissues than in adjacent normal ovarian tissues (Fig. 1C). Furthermore, Western blotting analysis on 10 pairs of tissue samples demonstrated the higher protein levels of AE2 in ovarian cancer tissues than that in adjacent normal tissues (Fig. 1D).

### Relationship between AE2 mRNA level and overall survival

Next, we examined the relationship between AE2 mRNA level and overall survival of patients with ovarian cancer. The patients were subdivided into lower expression group and higher expression group according to the median value of mRNA expression. Kaplan-Meier analysis demonstrated that high-level AE2 expression was correlated with shorter overall survival (median survival, 17.0 months vs 37.5 months for high and low level of AE2 mRNA in tumors, respectively; *P* < 0.01; Fig. 1E). These data emphasized the close association between AE2 expression and prognosis in patients with ovarian cancer.

### Knocking down AE2 inhibits ovarian cancer cell proliferation and induces G1-phase arrest

**Figure 2:**
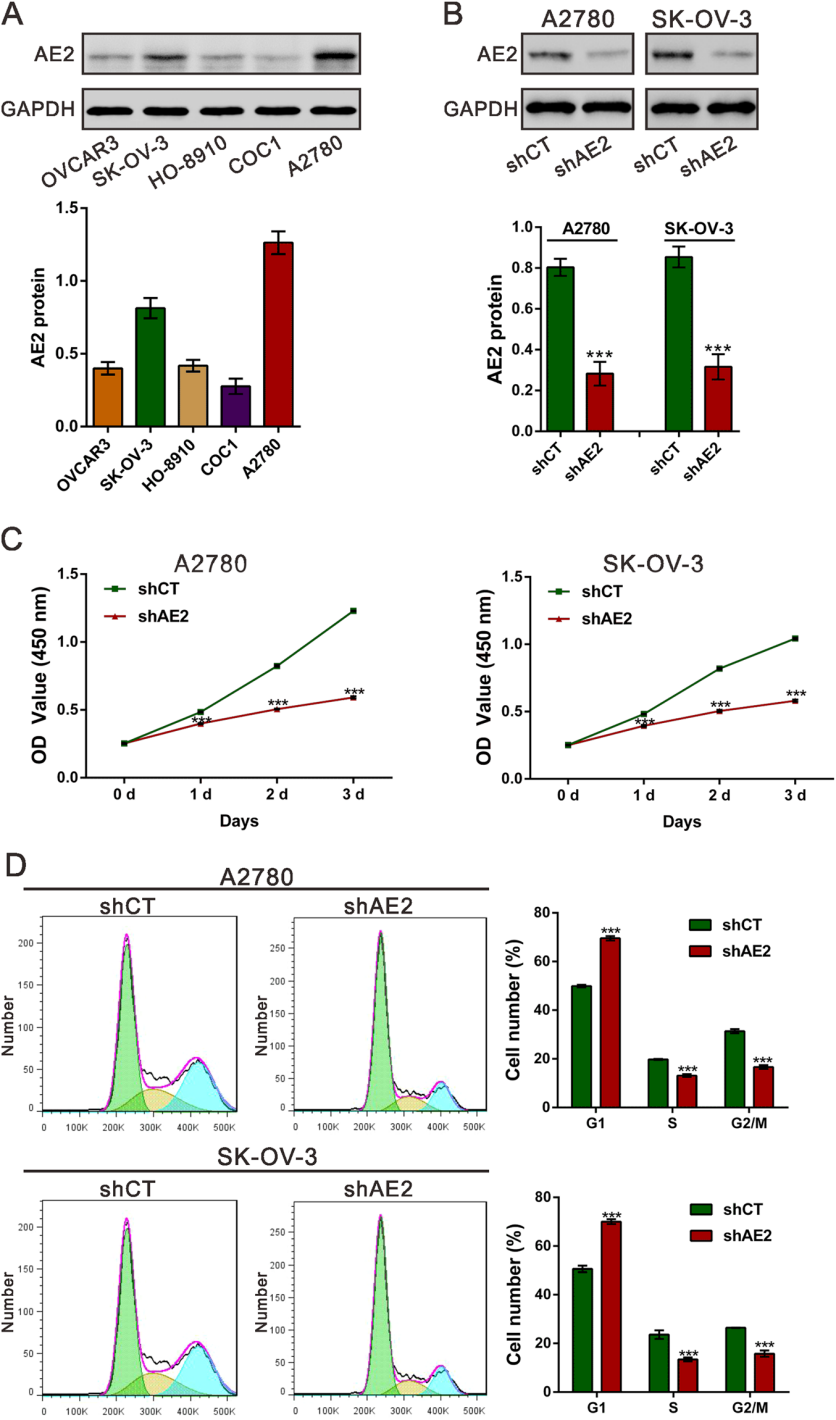
Knockdown AE2 inhibited ovarian cancer cell proliferation and blocked cell cycle progression. (**A**) Western blot analysis of AE2 expression in different ovarian cancer cell lines. The bar chart showed the ratio of AE2 protein to GADPH. (**B**) Knockdown efficiency was determined by Western blot analysis in A2780 and SK-OV-3 cells. (**C**) Knockdown AE2 significantly reduced the proliferative capacities of A2780 and SK-OV-3 cells, as determined by CCK-8 assays. (**C**) Knockdown AE2 induced cell arrest in G1 phase. Three independent experiments were performed, and data were represented as mean ± SD. ***P < 0.001 versus shCT.

Because AE2 was up-regulated in human ovarian cancer tissues, we next explored its biological role. Firstly, we detected the protein expression of AE2 in diverse ovarian cancer cell lines, including OVCAR3, SK-OV-3, HO-8910, COC1 and A2780. Higher protein expression of AE2 was observed in A2780 and SK-OV-3 cells (Fig. 2A). Next, we knocked down AE2 expression by short-hairpin RNA (shRNA) in A2780 and SK-OV-3 cells (Fig. 2B) and Cell Count Kit-8 (CCK-8) assay was performed to assess the capability of cell proliferation. As shown in Fig. 2C, shRNA-mediated knockdown of AE2 inhibited A2780/SK-OV-3 cell proliferation. We then examined whether knocking down AE2 inhibits cell cycle progression by cell cycle distribution analysis. Flow cytometric analysis revealed that shAE2 caused a significant increase in the percentage of cells in G1 phase (A2780: 69.54 ± 0.89% in A2780 AE2-knockdown stable cells [shAE2] vs 49.90 ± 0.55% in control stable cells [shCT]; SK-OV-3: 70.01 ± 1.00% in shAE2 cells vs 50.66 ± 1.33% in shCT cells), with a concomitant decrease in the percentage of cells in S and G2/M phase (Fig. 2D).

### Silencing of AE2 suppresses tumorigenesis of ovarian cancer cells *in vivo*

**Figure 3:**
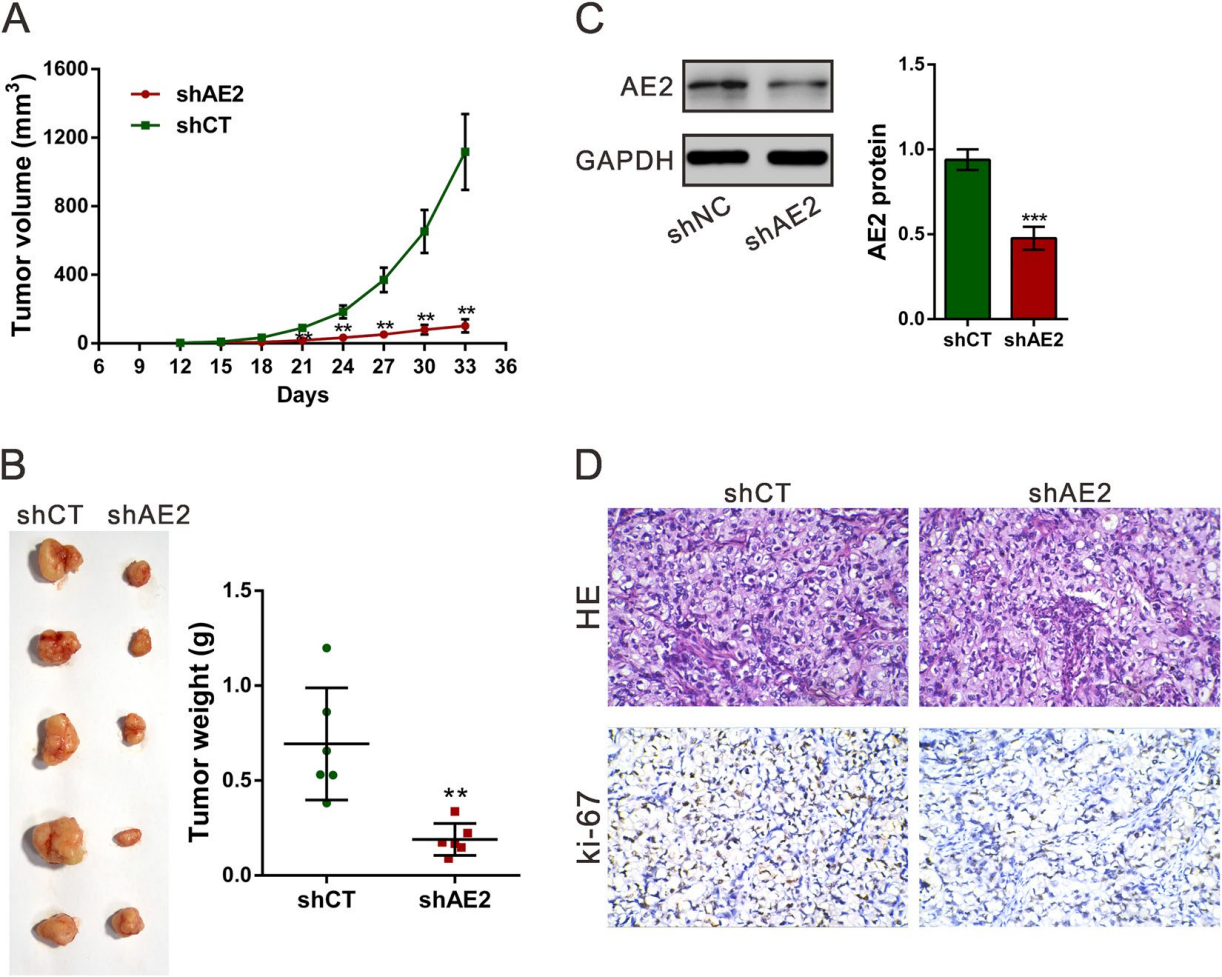
Knockdown AE2 inhibited ovarian cancer cells proliferation *in vivo*. A2780 stably transfected with shAE2 or shCT were subcutaneously inoculated into nude mice (**A**) The tumor size was monitored every three days. (**B**) Mice were sacrificed and the tumors were isolated after 33 days. (**C**) AE2 protein expression in xenograft was measured by Western blot analysis. Representative blot and quantification of western blot were shown. (**D**) Transplanted tumors with H&E staining and Ki-67 immunostaining. Magnification: 200× . **P < 0.01, ***P < 0.001 versus shCT.

To confirm the growth inhibitory effect of AE2-shRNA *in vivo*, A2780 shAE2 or shCT cells were subcutaneously injected into nude mice. As illustrated in Fig. 3A, xenograft tumors formed by shAE2 cells grew much slower than those by shCT cells. The weight of AE2 silenced tumors was much lighter than control tumors at 33days after cell inoculation (Fig. 3B). Western blot analysis revealed that AE2 protein was significantly decreased in tumors formed from shAE2 cells (Fig. 3C). Further, compared with that in NC-ones, Ki-67-positive cells were also significantly reduced in tumors formed from shAE2 cells (Fig. 3D). These results demonstrated the inhibitory effects of AE2 knockdown on the tumorigenesis of ovarian cancer cells *in vivo*.

### AE2-associated biological pathways in ovarian cancer

**Figure 4:**
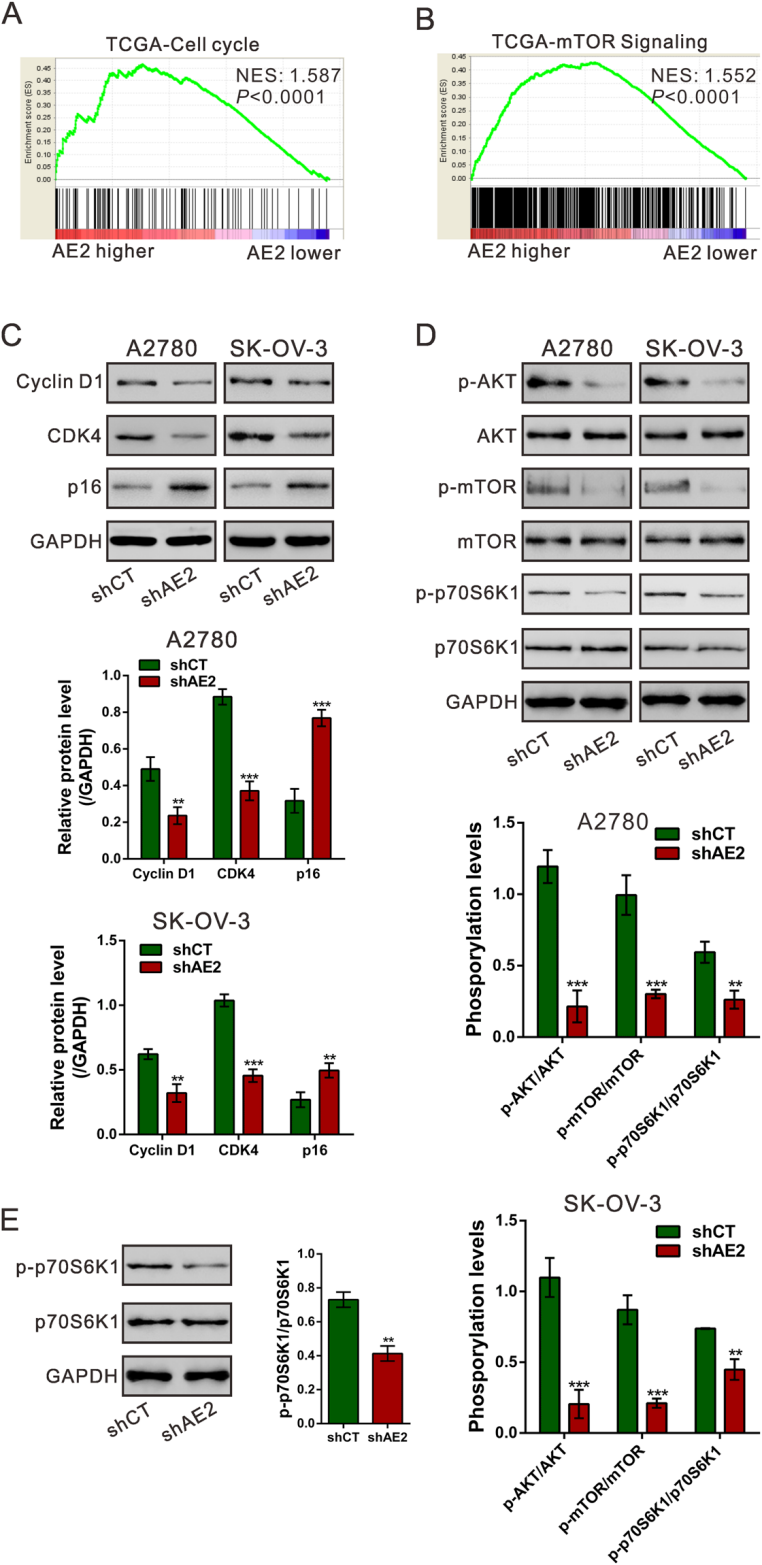
Mechanisms of AE2 exerts its function. (**A**,**B**) GSEA analysis in ovarian cancer patients with higher AE2 expression versus lower AE2 expression based on TCGA dataset. NES, normalized enrichment score. Cell cycle and mTOR signaling pathways have the strongest association with AE2-higher expression. (**C**,**D**) Protein levels of Cyclin D1, CDK4, p16, p-AKT, AKT, p-mTOR, mTOR, p-p70S6K1 and p70S6K1 were determined by Western blot. (**E**) The expression of p-p70S6K1 and-p70S6K1 in xenograft tumors was measured by Western blot. **P < 0.01, ***P < 0.001 versus shCT.

To probe the AE2-associated pathways in ovarian cancer, we performed GSEA using RNA-sequencing data from the TCGA ovarian cancer cohort. Cell cycle and mTOR signaling pathway were found strongly associated with AE2 expression (Fig. 4A and B). Cyclin D1 and CDK4 are key regulators of G1/S transition, whereas p16 is a well-known inhibitor for CDK4^21^. p70S6K1 is an important downstream effector of mTOR signaling^22^. AKT functions as a critical upstream regulator of mTOR signaling^23^. To validate the GSEA results, we then detected the levels of Cyclin D1, CDK4, p16, AKT, p-AKT, mTOR, p-mTOR, p70S6K1 and p-p70S6K1 in ovarian cancer cells with AE2 silenced. Consistent with the results of cell cycle analysis, AE2 knockdown in both A2780 and SK-OV-3 cells caused a notable decrease in the protein levels of Cyclin D1 and CDK4, and a remarkable increase in p16 protein expression (Fig. 4C). AE2 knockdown had no effects on the protein expression of AKT, mTOR or p70S6K1, but significantly reduced their phosphorylation levels (Fig. 4D). p-p70S6K1 was also decreased in xenograft formed from AE2 silenced cells (Fig. 4E).

### AE2 activated mTOR-p70S6K1 signaling

**Figure 5:**
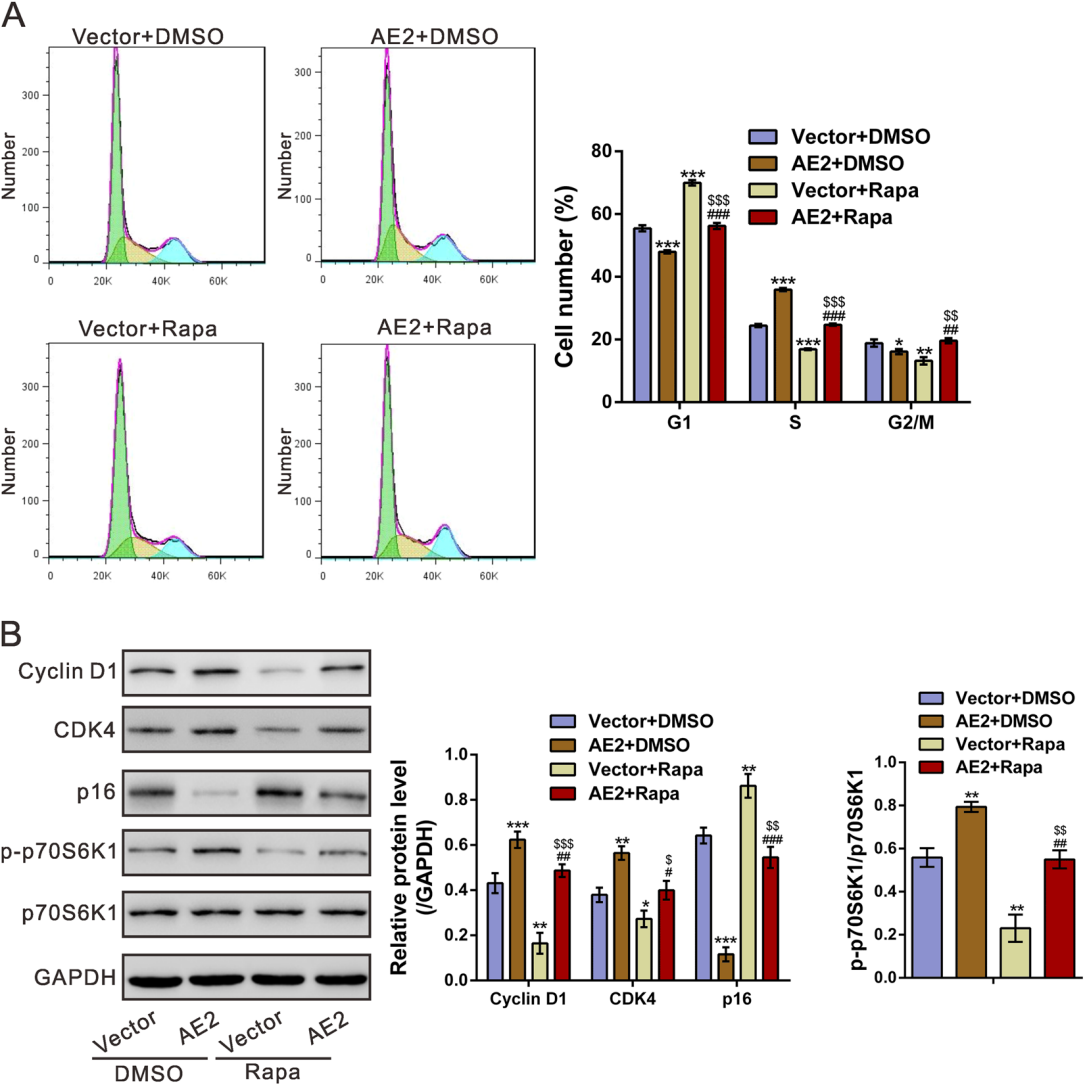
AE2 overexpression suppressed the effects of rapamycin on cell cycle progression. OVCAR3 cells were infected with pLVX-puro (Vector) or pLVX-puro /AE2 lentivirus, and then cultured for 36 h after infection. The cells were treated with DMSO or 10 μM rapamycin (Rapa) for 12 h. (**A**) The cell cycle progression was analyzed. Overexpression of AE2 reversed the effects of rapamycin on G1 arrest in OVCAR3 cells. (**B**) Protein levels of Cyclin D1, CDK4, p16, p-p70S6K1 and p70S6K1 were determined by Western blot. *P < 0.05, **P < 0.01, ***P < 0.001 versus Vector + DMSO; ^#^ *P* < 0.05, ^##^ *P* < 0.01, ^###^ *P* < 0.001 versus AE2 + DMSO; ^$^ *P* < 0.05, ^$$^ *P* < 0.01, ^$$$^ *P* < 0.001 versus Vector + Rapa.

It has been reported that inhibition of the mTOR-p70S6K1 activity by rapamycin induces the G1 cell cycle arrest in ovarian cancer cells^24^. In order to further confirm the involvement of mTOR-p70S6K1 signaling, OVCAR3 cells were overexpressed with AE2 and treated with 10 μM rapamycin. The cell cycle transition was significantly promoted by AE2 overexpression, but inhibited by rapamycin treatment. Moreover, the effects of rapamycin on cell cycle arrest was impaired by ectopic expression of AE2 (Fig. 5A). The expression of Cyclin D1, CDK4, p16, p70S6K1 and p-p70S6K1 was also detected by Western blotting (Fig. 5B). Rapamycin significantly reduced the levels of Cyclin D1, CDK4 and p-p70S6K1, and increased p16 expression. Such effects were weaken by AE2 overexpression. Thus, these data suggested that AE2 may promotes ovarian cancer cell cycle progression by activating mTOR-p70S6K1 pathway.

## Discussion

Previous studies have linked AE2 to the development of hepatocellular carcinoma^15, 16^, colon cancer^17^, gastric cancer^18, 19^ and bladder cancer^20^. In the current study, AE2 was identified as an up-regulated protein in ovarian cancer tissues compared with that in adjacent normal tissues. We demonstrated that AE2 expression was increased in ovarian cancer tissues and its expression was probably correlated with poor overall survival of patients. Our study, together with previous reports, supports the notion that AE2 plays critical roles in carcinogenesis. Knockdown of AE2 in A2780 and SK-OV-3 cells inhibited cell growth and cell cycle progression *in vitro* and *in vivo*, demonstrating that AE2 overexpression was functionally significant.

Further, we tried to investigate the mechanisms by which AE2 exerts its function. GSEA analysis based on TCGA OV dataset indicated that AE2 expression was associated with the cell cycle process and mTOR pathway. Dysregulated cell cycle progression, which is frequently observed in human malignancies, gives rise to aberrant cell proliferation. Here, AE2 knockdown remarkably decreased Cyclin D1 and CDK4 (key regulators of G1/S transition), but increased p16 (an inhibitor of CDK4^21^), which was consistent with the results of cell cycle distribution analysis. A recent study has showed that AE1 can bind with p16 and sequestrate p16 in the cytoplasm, thus plays a critical role in the pathogenesis of gastric and colonic cancer^14^. Our data revealed that AE2 affects p16 in a manner different from AE1 although detailed mechanism is needed further study. Furthermore, the mTOR/p70S6K1 pathway participates in the regulation of cell growth^25, 26^. It has been reported that mTOR pathway regulates the transcription, translation and stability of Cyclin D1 in various types of cancer cells^27–29^. mTOR is phosphorylated at Ser2448 by the PI3 kinase/AKT signaling pathway^23^. The phosphorylation of AKT and mTOR may be abnormally regulated in ovarian cancer^30^. Here, we confirmed that p-p70S6K1 was reduced in ovarian cancer cells with AE2 silenced, but increased in ovarian cancer cells with AE2 overexpression. p-p70S6K1 was also decreased in xenograft formed from AE2 silenced cells (Fig. 4E), which verified that the loss of AE2 led to lesser tumorigenesis through this pathway *in vivo*. Further, the induction effects of mTOR inhibitor, rapamycin on cell cycle G1 phase arrest in ovarian cancer cells was impaired by AE2 overexpression, which suggested AE2 might promote cell cycle progression partially by activating mTOR/p70S6K1 pathway. The protein expression of Cyclin D1 was higher in cells with higher phosphorylation levels of p70S6K1. mTOR activity is known to be affected by cell osmotic perturbations and be critical to cell volume control^31^. Ion transporters, including AE2, are involved in the regulation of cell volume^32^. Here, we assessed cell volume changes after AE2 knockdown by flow cytometry. In AE2 silenced A2780 cells, cell volume was decreased, which may lead to the inactivation of mTOR/p70S6K1 signaling pathway (Fig. S1). Although it remains to be determined the exact mechanisms by which AE2 regulates this pathway, these data suggested that AE2 might play a pivotal role in regulation of the cell cycle process through mTOR/p70S6K1 activation.

In summary, AE2 was overexpressed in ovarian cancer samples, and up-regulated AE2 expression can activate the mTOR/p70S6K1 pathway, promoting ovarian cancer cell growth. Our data identify AE2 as a potential novel target against ovarian cancer.

## Materials and Methods

### Tissue samples

The study was approved and performed in accordance with the guideline of the independent Ethics Committee of Seventh People’s Hospital and Fudan University Shanghai Cancer Center. A total of 100 patients undergoing resection of the primary ovrian cancer at Seventh People’s Hospital and Fudan University Shanghai Cancer Center were enrolled in this study. Clinical follow-up was conducted up to 60 months. Written informed consent was obtained from all patients. Both the ovarian cancer tissues and normal tissues underwent strict quality control measures before use. Pathology specimens were formalin-fixed paraffin-embedded (FFPE), hematoxylin and eosin (H&E)-stained, and examined by two indepent pathologists. Cancer cells with epithelial morphology were not observed in collected normal ovarian tissues. Collected tumor tissues and matched adjacent normal tissues were snap-frozen in liquid nitrogen and stored at −80 °C until used.

### Tandem mass tag (TMT) labeling and LC-MS/MS analysis

Protein was extracted from tumor (C1–C5) and adjacent normal tissues (N1-N5) by using protein extraction kit (Beyotime, Shanghai, China) and quantified by Bradford Protein quantitative kit (Beyotime) according to the manufacturer’s instructions. Protein from C1–C5 (200 μg /each sample) were combined as Sample C, while protein from N1-N5 (200 μg /each sample) were combined as Sample N. To remove detergent that may interfere with subsequent LC-MS/MS analysis, Sample C and Sample N were precipitated with acetone. After digestion with trypsin, Sample C was labeled with TMT-129, TMT-130 and TMT-131, while Sample N was labeled with TMT-126, TMT-127 and TMT-128. Equal amount of Sample C and Sample N were then combined, separated into 15 componets by high-performance liquid chromatography (HPLC), and subjected to MS/MS analysis.

### The cancer genome atlas data analyses

Gene mutation and copy-number variations of AE1 (SLC4A1), AE2 (SLC4A2) and AE3 (SLC4A3) across ovarian cancer datasets were analyzed by the cBioportal for Cancer Genomics (www.cbioportal.org/). The AE2 mRNA expression levels in 8 normal tissues and 568 ovarian cancer tissues were examined in the ovarian serous cystadenocarcinoma (OV) dataset (The Cancer Genome Atlas, TCGA https://tcga-data.nci.nih.gov/tcga/).

### Reagents and cell culture

The mTOR inhibitor, rapamycin, was purchased from Calbiochem (San Diego, CA, USA). Propidium iodide (PI) was from Sigma. Anti-AE2 from Affinity (Cincinnati, OH, USA).The antibodies against p16, CDK4 and Cyclin D1 were from Santa Cruz Biotechnology (Santa Cruz, CA), and the antibodies against p-AKT (Ser^473^), AKT, p-mTOR (Ser^2448^), mTOR, p70S6K1, phospho-p70S6K1 (Thr^389^) and GAPDH were from Cell Signaling Technology (Beverly, MA). The horseradish peroxidase (HRP)-conjugated anti-rabbit IgG and anti-mouse IgG were from Beyotime. The human ovarian cancer cell lines OVCAR3, SK-OV-3, HO-8910, COC1 and A2780 (American Type Culture Collection; Rockville, MD, USA) were maintained in RPMI 1640 (Hyclone, Logan, UT, USA), supplemented with 10% fetal bovine serum (FBS, Hyclone) and penicillin/streptomycin, and cultured at 37 °C in a 5% CO_2_ incubator.

### Quantitative reverse transcription PCR (qPCR)

The total RNA was isolated from tissue samples or cultured cells using the TRIzol reagent (Life Technologies, Grand Island, NY, USA) according to the manufacturer’s instructions. Total RNA (1 μg) was reverse-transcribed into first-strand complementary DNA (cDNA) with oligo-dT primers and the SuperScript II Reverse Transcriptase (Invitrogen). The qPCR was performed with SYBR Green PCR kit (Thermo Fisher Scientific, Rockford, IL, USA) using an ABI 7300 Real-Time PCR machine (Applied Biosystems, Foster City, CA, USA). qPCR experiments were performed in triplicate. The average threshold cycle (Ct) value of AE2 was normalized to the average Ct value of glyceraldehyde-3-phosphase dehydrogenase (GAPDH). The specific primers used in the qPCR were as follows: for SLC4A2 (AE2), 5′-GCACCGCAGCTACAACCTTC-3′ and 5′-AGCGTCTGGGCCTCAATCTC-3′; and for GAPDH, 5′-CACCCACTCCTCCACCTTTG-3′ and 5′-CCACCACCCTGTTGCTGTAG-3′.

### Western blotting analysis

The tissue samples or cultured cells were lysed on ice for 30 min in RIPA buffer (Beyotime), supplemented with protease inhibitor cocktail (Roche Applied Science, Indianapolis, IN, USA). After centrifugation at 13,000 rpm for 15 min, the supernatant was harvested and the protein concentration was determined by using BCA assay kit (Thermo Fisher Scientific). Protein extracts were separated by sodium dodecyl sulfate–polyacrylamide gel electrophoresis (SDS–PAGE) and transferred to nitrocellulose membrane. After blocking with 5% nonfat dry milk, the membranes were incubated with primary antibodies at 4 °C overnight, followed by the incubation with the appropriate HRP-conjugated IgG. The specific proteins in the blots were visualized by using enhanced chemiluminescence (ECL, Millipore, Bredford, USA). The densities of the protein bands were quantified using Image J software (NIH, USA).

### Plasmid constructs, short-hairpin RNA-mediated knockdown, viral infection and stable cell line establishment

Full-length AE2 was inserted into lentivirus pLVX-puro vector (Clontech, Palo Alto, CA, USA) within EcoRI/BamHI site by Genewiz (Suzhou, China). shRNA targeting AE2 (shAE2) or scramble shRNA (shCT) were cloned into pLKO.1 (Addgene).

For the generation of lentiviral particles, targeted viral plasmid, psPAX2 and pMD2G were used to transfect 293 T cells with Lipofectamine2000 (Life Technologies). At 48 h after transfection, the supernatants containing viral particles were filtered using 0.45 μm filters.

A2780 and SK-OV-3 cells were infected with shAE2 or shCT viral supernatants, and OVCAR3 were infected with AE2-overexpression virus or control virus. Viral transduction was performed in the present of 10 μg/ml Polybrene (Sigma-Aldrich, St. Louis, MO, USA). At 48 h after viral transduction, AE2 expression was assessed by Western blotting analysis.

A2780 AE2-knockdown stable cell lines (shAE2) or control stable cell lines (shCT) were generated by puromycin (Sigma-Aldrich) selection and used for *in vivo* tumorigenicity assay.

### Cell proliferation assays

Cells were seeded in 96-well plates (1000–1500 cells /well), infected with indicator virus and incubated for 1, 2 or 3 days. Cell proliferation was detected with the Cell Count Kit-8 (CCK-8, Beyotime) following the manufacturer’s instructions. Briefly, at indicated time point, CCK8 solution was added to each well and incubated for 1 h. Optical density (OD) at 450 nm was detected with a microplate reader (Bio-Rad). All samples were assayed in triplicate and the assays were repeated three times.

### Cell cycle analysis

The cell cycle distribution was examined by propidium iodide (PI) staining and flow cytometry analysis. Briefly, cells were collected, washed twice with PBS and fixed with ice-cold 70% ethanol overnight at −20 °C. The fixed cells were then washed twice with PBS and incubated with staining buffer (0.1% Triton X-100, 50 μg/ml RNase and 50 μg/ml PI) for 15 min at 37 °C. The cell cycle was examined using an Accuri C6 flow cytometer (BD Biosciences, San Jose, CA, USA). At least 10^4^ cells were collected for each sample. The percentage of cells in each phase (G0/1, S, and G2/M) was determined by FlowJo software (version 7.6.1, Tree Star, Ashland, OR, USA).

### Tumorigenicity assay

The animal experiments were approved and performed according to the guidelines of IACUC committee at Shanghai University of Traditional Chinese Medicine. Nude mice (SLAC laboratory animal Center, Shanghai, China), 5 to 7 weeks old, were subcutaneously injected into the right flank with A2780 AE2-knockdown stable cell lines (shAE2) or control stable cell lines (shCT) (2 × 10^6^ in 100 μl of PBS). Caliper measurements of the largest (L) and the smallest diameter (D) of the tumors were done every three days to estimate tumor volume (V) using the following formula: V = 1/2 × L × D^2^. After 33 days, the mice were sacrificed, and the xenograft tumors were recovered and weighed. Hematoxylin/eosin (H&E) staining according to standard protocols. To assess the proliferating cells in the xenograft tumors, Ki-67 immunohistochemistry staining (Abcam) was performed. AE2 expression in the xenograft tumors was analyzed by Western blotting.

### Gene Set Enrichment Analysis (GSEA)

In this study, ovarian cancer cohort obtained from The Cancer Genome Atlas (TCGA, https://tcga-data.nci.nih.gov/tcga/) was analyzed by GSEA as previously described^33^. GSEA was performed using the GSEA software, Version 2.0.1, obtained from the Broad Institute (http://www.broad.mit.edu/gsea; ref. 20) as previously described^34–36^. GSEA version 2.0 software was from the Broad Institute at MIT. The nominal P value and normalized enrichment score (NES) was used to sort the pathways associated with AE2-higher expression.

### Data analysis

All experiments were independently repeated three times. The results are presented as the means ± SD. One-way analysis of variance (ANOVA) was performed to calculate the statistical significance of difference. Overall survival was analyzed by the Kaplan-Meier survival curves with log-rank nonparametric test. P-values < 0.05 were considered statistically significant.

## Electronic supplementary material

Table S1

Table S2

Figure S1
