# Supplementary material for: Knockdown of anion exchanger 2 suppressed the growth of ovarian cancer cells via mTOR/p70S6K1 signaling
Source: Sci Rep. 2017 Jul 25;7:6362. doi: 10.1038/s41598-017-06472-w (PMC5526859; doi:10.1038/s41598-017-06472-w)
Supplement: Supplementary file 3 — Figure S1 [file 41598_2017_6472_MOESM3_ESM.pdf]

Knockdown of anion exchanger 2 suppressed the growth of ovarian cancer cells via mTOR/p70S6K1 signaling

Li-Jun Zhang[1#], Renquan Lu[2,3#], Ya-Nan Song[1], Jian-Yong Zhu[1], Wei Xia[4], Miao Zhang[1], Zhi-Yi Shao[1],Yan Huang[3,5], Yuqi Zhou[3,5], Hongqin Zhang[2,3] , Lin Guo[2,3*], Meiqin Zhang[3,5*], Hong Zhang[1*]

[1]Central Laboratory, Seventh People's Hospital of Shanghai University of Traditional Chinese Medicine, Shanghai 200137, China

[2]Department of Clinical Laboratory, Fudan University Shanghai Cancer Center, Shanghai 200032, China

[3]Department of Oncology, Shanghai Medical College, Fudan University, Shanghai 200032, China

[4]Department of Nuclear Medicine, Seventh People's Hospital of Shanghai University of Traditional Chinese Medicine, Shanghai 200137, China

[5]Department of Gynecological Oncology, Fudan University Shanghai Cancer Center, Shanghai 200032, China

[#]Contributed equally to this work

*Corresponding to: Hong Zhang (E-mail: hzhang51@126.com), 358 Datong Road, Shanghai 200137, China; Lin Guo (E-mail: guolin500@hotmail.com) and Meiqin Zhang (E-mail: zhangmq67@126.com), 270 DongAn Road, Xuhui District, Shanghai 200032, China.

Materials and Methods

Cell volume analysis

Cell volume was investigated by fluorescence-activated cell sorter (FACS) analysis. A2780 cells were collected, resuspended in RPMI 1640, and directly measured by forward scatter on an Accuri C6 flow cytometer (BD Biosciences, San Jose, CA, USA).

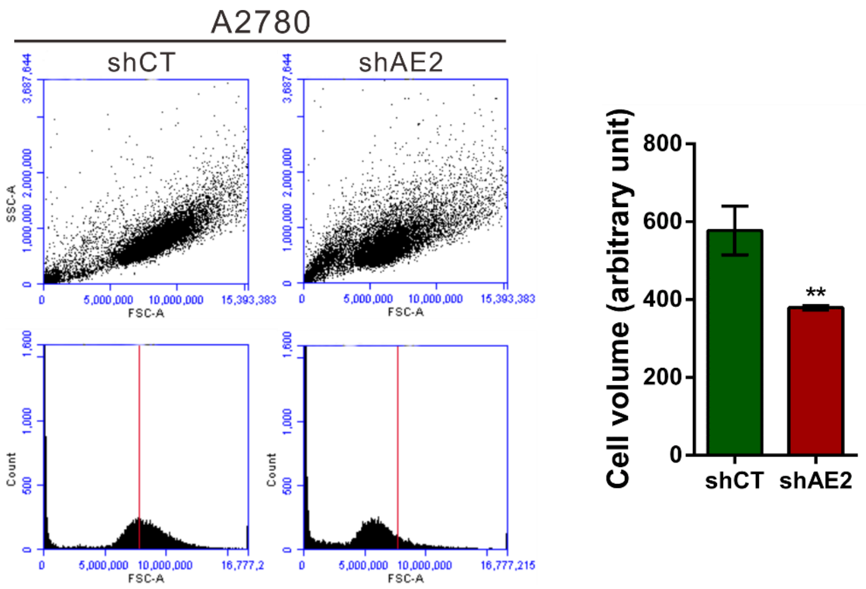

Figure S1. Cell volume analysis by flow cytometry. Dot blots of forward-scatter (FSC) vs. sideward-scatter (SSC), and FACS histograms were shown. The arbitrary units on the x-axis (FSC) in FACS histograms are proportional to cell size. The shifting of peaks implies alterations in cell volume (10,000 cells, n=3). ***P*<0.01 versus shCT.
